# Novel FXR agonist nelumal A suppresses colitis and inflammation-related colorectal carcinogenesis

**DOI:** 10.1038/s41598-020-79916-5

**Published:** 2021-01-12

**Authors:** Tsuneyuki Miyazaki, Yohei Shirakami, Taku Mizutani, Akinori Maruta, Takayasu Ideta, Masaya Kubota, Hiroyasu Sakai, Takashi Ibuka, Salvatore Genovese, Serena Fiorito, Vito Alessandro Taddeo, Francesco Epifano, Takuji Tanaka, Masahito Shimizu

**Affiliations:** 1 Department of Gastroenterology, Gifu University Graduate School of Medicine, 1-1 Yanagido, Gifu, 501-1194 Japan; 2 Department of Pharmacy, D’Annunzio University of Chieti–Pescara, 66100 Chieti Scalo, Italy; 3 Department of Pathological Diagnosis, Gifu Municipal Hospital, Gifu, 500-8513 Japan

**Keywords:** Gastrointestinal diseases, Cancer prevention

## Abstract

FXR is a member of the nuclear receptor superfamily and bile acids are endogenous ligands of FXR. FXR activation has recently been reported to inhibit intestinal inflammation and tumour development. This study aimed to investigate whether the novel FXR agonist nelumal A, the active compound of the plant *Ligularia nelumbifolia*, can prevent colitis and colorectal carcinogenesis. In a mouse colitis model, dextran sodium sulfate-induced colonic mucosal ulcer and the inflammation grade in the colon significantly reduced in mice fed diets containing nelumal A. In an azoxymethane/dextran sodium sulfate-induced mouse inflammation-related colorectal carcinogenesis model, the mice showed decreased incidence of colonic mucosal ulcers and adenocarcinomas in nelumal A-treated group. Administration of nelumal A also induced tight junctions, antioxidant enzymes, and FXR target gene expression in the intestine, while it decreased the gene expression of bile acid synthesis in the liver. These findings suggest that nelumal A effectively attenuates colonic inflammation and suppresses colitis-related carcinogenesis, presumably through reduction of bile acid synthesis and oxidative damage. This agent may be potentially useful for treatment of inflammatory bowel diseases as well as their related colorectal cancer chemoprevention.

## Introduction

Colorectal cancer (CRC) is a significant and increasing health care problem worldwide. CRC is one of the most critical complications of inflammatory bowel disease (IBD) including ulcerative colitis and Crohn’s disease^1^. IBD has become a significant disease with an increasing incidence, especially in newly industrialized countries. Therefore, the number of patients with IBD-related CRC is anticipated to continue to increase^2^. Scientific evidence has indicated that chronic inflammation and oxidative stress in the large intestine are crucial factors in promoting colorectal tumourigenesis. Additionally, increased fecal excretion of bile acids is associated with an elevated incidence of CRC. However, the mechanism by which bile acids contribute to colorectal cancer is not fully understood^3^.

Farnesoid X receptor (FXR) is a nuclear receptor highly expressed in the liver and the intestine. FXR is a bile acid-associated receptor and a key regulator of bile acid metabolism as well as lipid and glucose homeostasis^4^. To modulate bile acid homeostasis, FXR decreases bile acid synthesis and uptake and increases secretion in hepatocytes while reducing bile acid absorption in the enterocytes. FXR also plays a role in the intestinal barrier function and antibacterial defense. The activation of FXR can reduce inflammation and preserve the mucosal barrier in IBD^5^. Previous research has indicated that the expression levels of pro-inflammatory cytokines are increased in FXR-deficient mice^6^. In several rodent colon tumourigenesis models, FXR deficiency results in markedly increased sizes and numbers of colonic neoplasms^7^. These findings suggest that the upregulation of FXR expression and the activation of FXR signaling may be useful for the reduction of intestinal inflammation and in the prevention and treatment of CRC.

In recent years, FXR ligands have been shown to extend beyond bile acids to endogenous and exogenous compounds and natural and synthetic agents. The agonists of FXR are paid attention by researchers for various pathological condition^8–10^. Several FXR agonists, including the most widely used GW4064, have been employed in rodent colitis models, where they exert suppressing effects on intestinal inflammation^11^. Although GW4064 and its derivatives can be powerful analytical tools and treatment agents, their therapeutic effects are limited due to low bioavailability and adverse effects^12–14^. Among the chemically synthetic FXR agonists reported by Epifano et al.^15^, nelumal A, the active compound of the plant *Ligularia nelumbifolia*, exhibited an efficacy comparable to that of the endogenous FXR ligand, showing superior activity over that of chenodeoxycholic acid. Nelumal A also showed stronger activity compared to an FXR agonist auraptene which is reported to have anti-cancer, anti-inflammatory, and anti-oxidant properties as well as to suppress colon carcinogenesis in several rodent models^16–18^.

Herein, we aimed to examine the possible inhibitory effects of the novel FXR agonist nelumal A on colitis and inflammation-associated colon carcinogenesis, dextran sodium sulfate(DSS-induced colitis and azoxymethane (AOM) plus dextran sodium sulfate (DSS)-induced colitis and AOM plus DSS-induced colon carcinogenesis^19^. Using these two models, we have reported that various synthetic or natural compounds could effectively attenuate and suppress colitis-associated CRC development^20–24^. In the first experiment, we determined the effects of nelumal A on DSS-induced colorectal inflammation and the expression of inflammatory cytokines and antioxidative enzymes in mice. In the second experiment, we evaluated the impact of nelumal A on colitis-related colon carcinogenesis in mice.

## Results

### Experiment 1 (DSS-induced colitis model)

#### General observation

There was no significant difference in body weight in any of the five groups at the end of the experiment, as shown in Supplementary Table S1. No significant differences were observed in the liver weight or the large intestine length among the groups. Growth curves of the mice during the experiment are shown in Fig. S1a. During the experiment 1, there was no physical signs of toxicity or poor condition in any experimental mice. Histopathological examination revealed no changes suggesting toxicity by nelumal A administration to the organs including the liver, kidneys, pancreas, spleen, heart, and lungs.

#### Incidence and multiplicity of mucosal ulcer and grade of inflammation in the colon

The incidence and multiplicity of colonic mucosal ulcers and the colonic inflammation grade at the end of the experiment are listed in Table 1. The multitude of mucosal ulcers decreased with increasing nelumal A dose. A significant decrease was observed when comparing mice that received 400-ppm nelumal A with the mice that received DSS treatment without nelumal A (Group 1). Treatment with 400-ppm nelumal A significantly decreased inflammation grade in the colon.

**Table 1:** Incidence and multiplicity of colonic mucosal ulcer and grade of inflammation in the experiment 1.

Group no.	Treatment	No. of mice	Mucosal ulcer		Inflammation grade in the mucosa
			Incidence	Multiplicity^a^	
G1-1	DSS	5	5/5 (100%)	4.2 ± 1.5^b^	3.2 ± 0.4
G1-2	DSS/100 ppm nelumal A	5	5/5 (100%)	2.4 ± 1.1	2.2 ± 0.8
G1-3	DSS/400 ppm nelumal A	5	2/5 (40%)	0.8 ± 1.3^c^	1.2 ± 0.8^c^
G1-4	400 ppm nelumal A	5	0/5 (0%)	0	0
G1-5	No treatment	5	0/5 (0%)	0	0

^a^Number of lesions per mouse.

^b^Mean ± SD.

^c^Significantly different from group 1 by Tukey–Kramer multiple comparison test (*P* < 0.05).

#### Expression levels of inflammatory cytokine and anti-oxidant enzyme mRNAs in colonic mucosa

**Figure 1:**
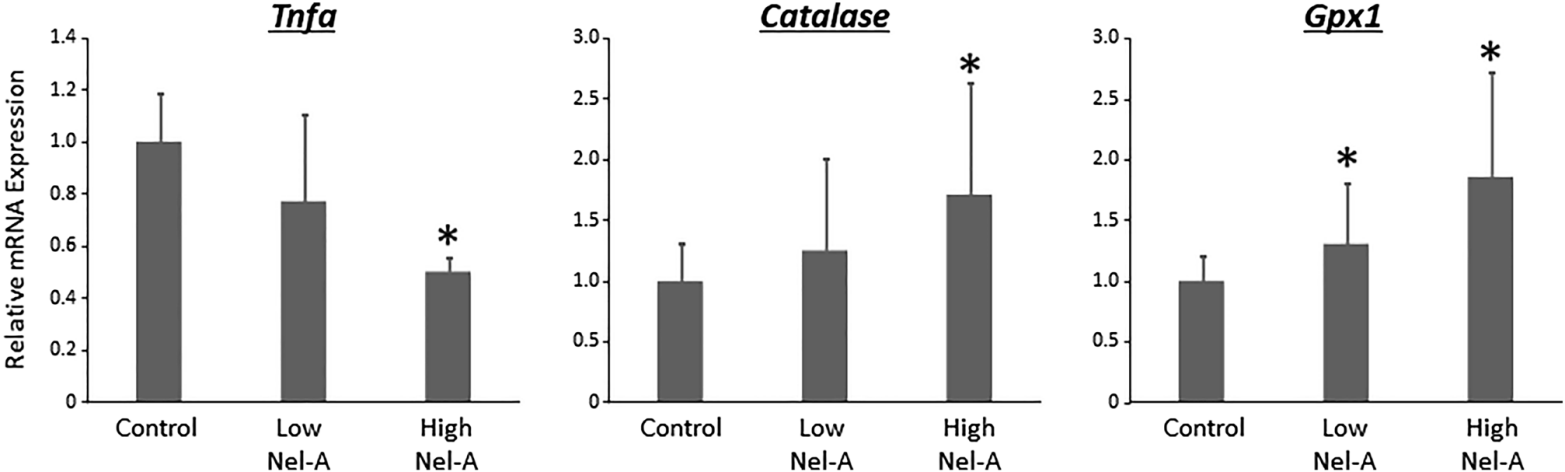
Effects of nelumal A on expression levels of TNF-α and anti-oxidant enzymes in the colon of colitis model mice. The mRNA expression levels of *Tnfa*, *Catalase*, and *Gpx1* in the colonic mucosa were measured by quantitative real-time reverse transcription PCR with specific primers. Parallel amplification of *18S* was used as the internal control. Each column represents the mean ± SD. Asterisk indicates statistically significant differences compared to DSS-treated group; *P* < 0.05. Statistical analyses were performed using one-way ANOVA followed by Tukey–Kramer multiple comparison test. n = 5. Nel-A, nelumal A.

Figure 1 shows the relative mRNA expression levels of tumour necrosis factor alpha (*TNF-α*), catalase, and glutathione peroxidase 1 (*Gpx1*) by real-time quantitative transcription PCR (qRT-PCR) analyses. TNF-α expression in the colonic mucosa was suppressed by nelumal A in a dose-dependent manner. Treatment with 400-ppm nelumal A caused a significant reduction in the expression of TNF-α as compared to that in the DSS-only control mice. The expression of the antioxidant enzymes *catalase* and *Gpx1* was increased by nelumal A treatment in a dose-dependent manner. Treatment with all doses of 400-ppm nelumal A significantly increased the expression of catalase and Gpx1, respectively, as compared to treatment with DSS-only in the control group.

### Experiment 2 (AOM/DSS-induced CRC model)

#### General observation

As shown in Supplementary Table S2, no significant differences were observed in body weight, relative liver weight, and length of the large intestine among the groups in Experiment 2. Body weight changes of the mice during the experiment are shown in Fig. S1b. During the experiment 2, there was no physical signs of toxicity or poor condition in any experimental mice. Histopathological examination revealed no changes suggesting toxicity by nelumal A administration in the liver, kidneys, pancreas, spleen, heart, and lungs.

#### Incidence and multiplicity of mucosal ulcer and dysplasia and grade of inflammation in the colon

Data on the incidence and multiplicity of colonic mucosal ulcers and colonic dysplasia at the end of the experiment are listed in Table 2. The incidence and multiplicity of mucosal ulcers decreased with nelumal A treatment. The decrease in mice receiving 400-ppm nelumal A was significant (*P* < 0.05). However, there was no significant difference in the incidence of colonic dysplasia. Compared to AOM/DSS-treated group, administration of high-dose nelumal A significantly decreased inflammation grade in the colon (Table S2).

**Table 2:** Incidence and multiplicity of colonic mucosal ulcer, dysplasia, adenoma and adenocarcinoma in the experiment 2.

Group no.	Treatment	No. of mice	Incidence				Multiplicity			
			Mucosal ulcer	Dysplasia	Adenoma	Adeno-carcinoma	Mucosal ulcer	Dysplasia	Adenoma	Adeno-carcinoma
G2-1	AOM/DSS	12	11/12 (92%)	11/12 (92%)	6/12 (50%)	6/12 (50%)	1.8 ± 0.9^b^	2.9 ± 1.9	1.2 ± 1.6	0.9 ± 1.3
G2-2	AOM/DSS/100 ppm nelumal A	12	7/12 (58%)	9/12 (75%)	6/12 (50%)	6/12 (50%)	1.0 ± 1.0	1.8 ± 1.5	1.0 ± 1.3	1.0 ± 1.3
G2-3	AOM/DSS/400 ppm nelumal A	12	5/12 (42%)^c^	9/12 (75%)	5/12 (42%)	1/12 (8%)^c^	0.7 ± 0.9^d^	1.9 ± 1.4	0.8 ± 1.2	0.1 ± 0.3
G2-4	400 ppm nelumal A	5	0/5 (0%)	0/5 (0%)	0/5 (0%)	0/5 (0%)	0	0	0	0
G2-5	AOM alone	5	0/5 (0%)	0/5 (0%)	0/5 (0%)	0/5 (0%)	0	0	0	0
G2-6	DSS alone	5	0/5 (0%)	0/5 (0%)	0/5 (0%)	0/5 (0%)	0	0	0	0
G2-7	No treatment	5	0/5 (0%)	0/5 (0%)	0/5 (0%)	0/5 (0%)	0	0	0	0

^a^Number of lesions per mouse.

^b^Mean ± SD.

^c^Significantly different from group 1 by Fisher’s exact probability test (*P* < 0.05).

^d^Significantly different from group 1 by Tukey–Kramer multiple comparison test (*P* < 0.05).

#### Incidence and multiplicity of colonic adenoma and adenocarcinoma

Data of the incidence and multiplicity of colonic tumours at the end of the experiment are listed in Table 2. Although there was no significant difference in the incidence of colonic adenoma, the incidence of adenocarcinoma was significantly lower in mice that received 400-ppm nelumal A than in mice that received AOM/DSS treatment.

#### Cell proliferation and apoptosis in colonic adenocarcinoma

**Figure 2:**
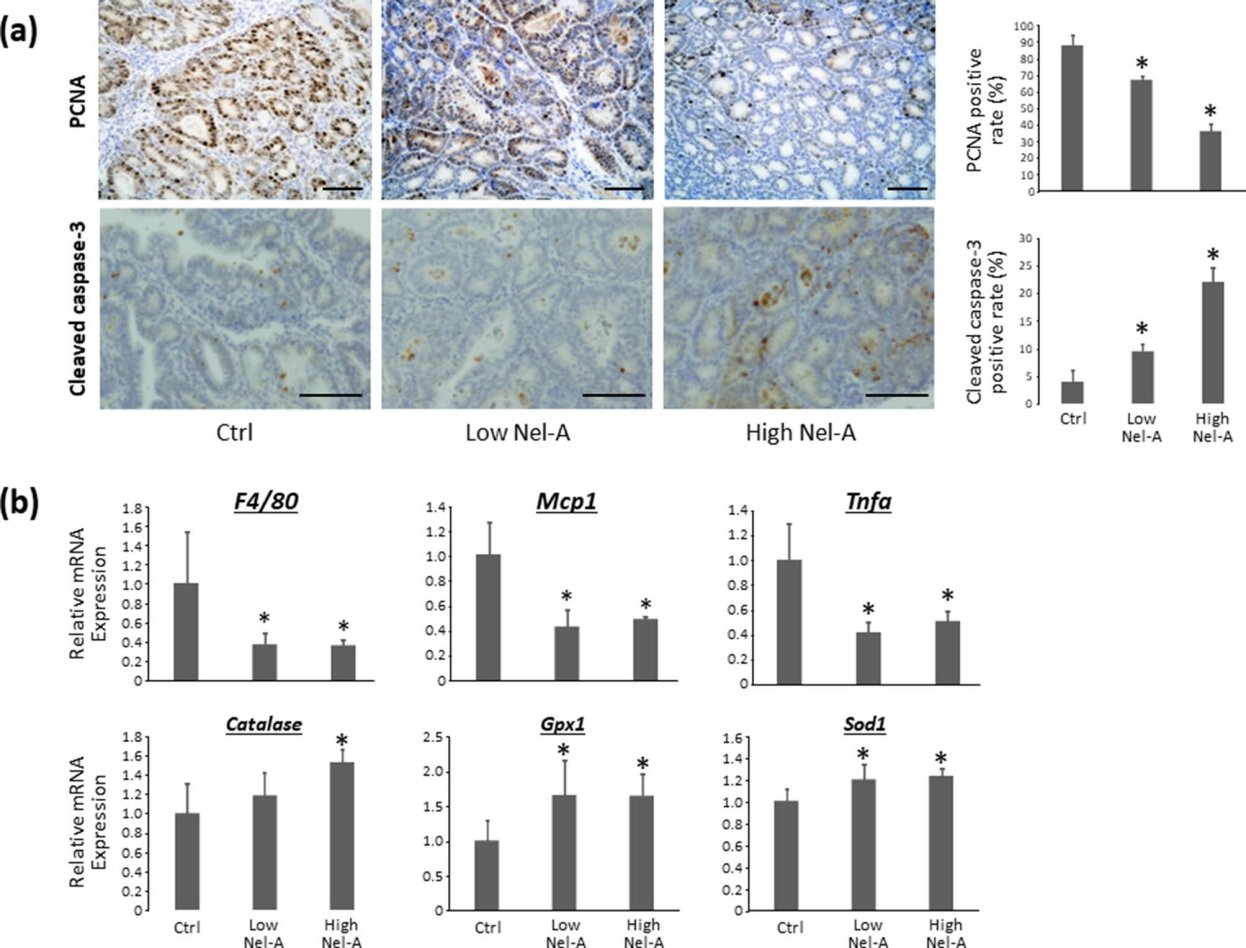
Effects of nelumal A on cellular proliferation and apoptosis in colorectal tumour tissues, and on inflammatory cytokines and anti-oxidant enzymes in colonic mucosa of AOM/DSS-treated mice. (**a**) Sections of the colon were stained with anti-PCNA or anti-cleaved caspase-3 antibodies. Representative photographs from each group are shown in the left panels. The positive cell indices, which were determined by counting positive cells, are shown in the right panels. (**b**) The mRNA expression levels of *F4/80*, *Mcp1*, *Tnfa*, *Catalase*, *Gpx1*, and *Sod1* in the colonic mucosa were measured by quantitative real-time reverse transcription PCR with specific primers. Parallel amplification of *18S* was used as the internal control. Scale bars, 100 μm. Each column represents the mean ± SD of triplicate assays. Asterisk indicates statistically significant differences compared to AOM/DSS group; *P* < 0.05. Statistical analyses were performed using one-way ANOVA followed by Tukey–Kramer multiple comparison test. n = 6. Ctrl, control. Nel-A, nelumal A.

To investigate the effect of nelumal A on cell proliferation and apoptosis at the end of the experiment, the expression of proliferating cell nuclear antigen (PCNA) and cleaved caspase-3 in colonic adenocarcinomas was analyzed immunohistochemically. As shown in Fig. 2a, PCNA-labeling indices of colonic adenocarcinomas in mice treated with 100- and 400-ppm nelumal A were markedly less than those of the AOM/DSS-treated group. In addition, the index was significantly lower in the group that received 400-ppm nelumal A than in the 100-ppm group. On the other hand, cleaved caspase-3-labeling indices of colonic adenocarcinomas in mice treated with 100- and 400-ppm nelumal A were markedly higher than that in the mice treated with AOM/DSS. Moreover, the cleaved caspase-3 index was significantly higher in the 400-ppm group than in the 100-ppm group.

#### Expression levels of inflammatory cytokine and anti-oxidant enzyme mRNAs in colonic mucosa

The relative mRNA expression levels of *TNF-α*, monocyte chemoattractant protein-1 (*Mcp1*), *F4/80* antigen, *catalase*, *Gpx1*, and *Sod1* genes in colonic mucosa by qRT-PCR analyses are shown in Fig. 2b. The expression of *TNF-α*, *Mcp1*, and *F4/80* in the colonic mucosa was found to be suppressed by nelumal A administration compared to that in the AOM/DSS group. Treatment with nelumal A also reduced the expression of interleukin 6, but the difference was not significant. The expression of the antioxidant enzymes catalase, *Gpx1*, and *Sod1* was increased by nelumal A treatment as compared to that in the AOM/DSS group. These results indicate that nelumal A could attenuate inflammation and oxidative stress in the colonic mucosa of experimental mice.

#### Effects of nelumal A on cell proliferation and barrier function in colonic mucosa

**Figure 3:**
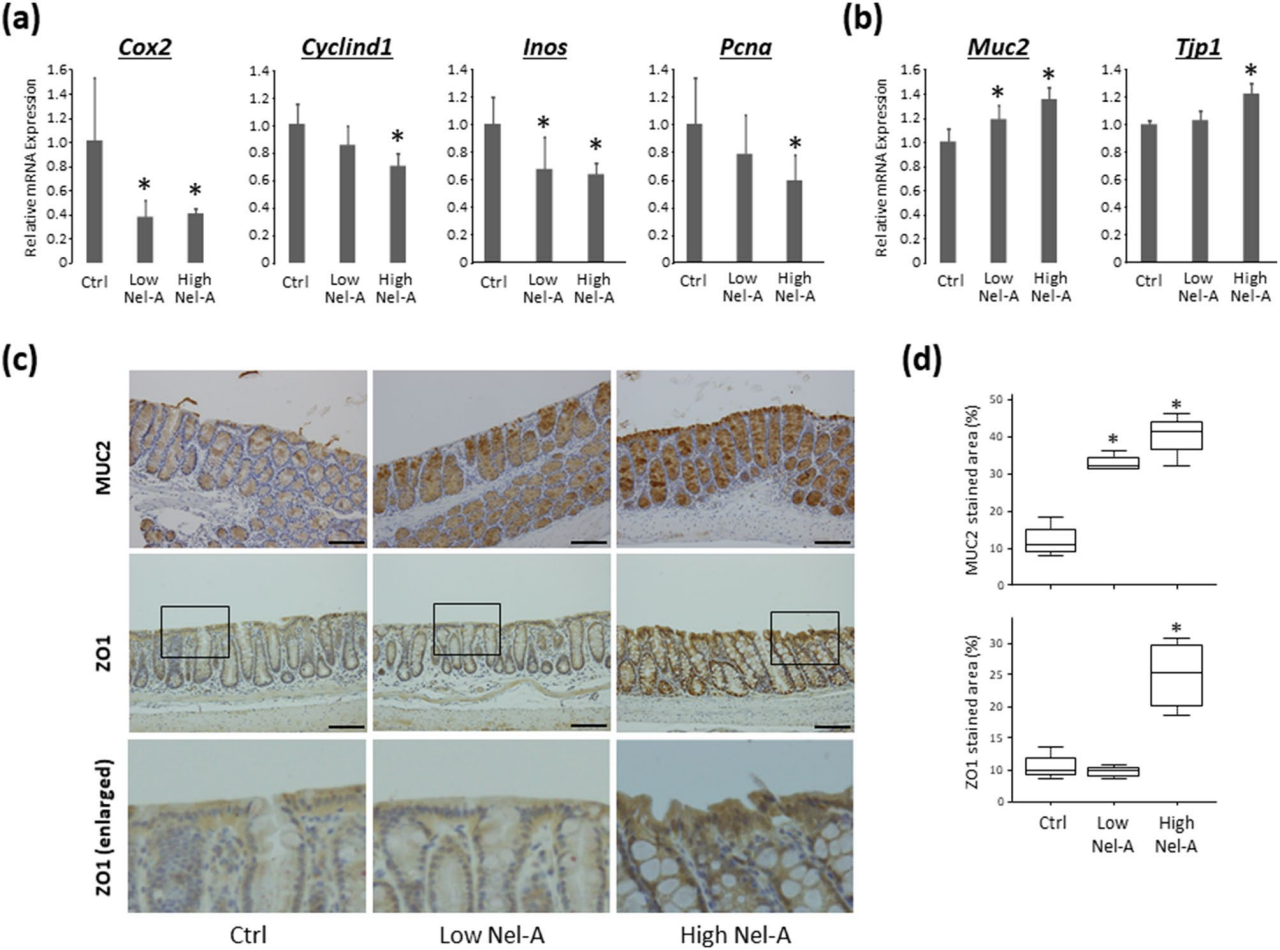
Effects of nelumal A on cell proliferation and barrier function in the colon of carcinogenesis model mice. The mRNA expression levels of Cox2, Cyclind1, Inos, Pcna (**a**), Muc2, and Tjp1 (**b**) in the colonic mucosa were measured by quantitative real-time reverse transcription PCR with specific primers. Parallel amplification of 18S was used as the internal control. Each column represents the mean ± SD. Statistical analyses were performed using one-way ANOVA followed by Tukey–Kramer multiple comparison test. n = 6. (**c**) Immunohistochemical analyses for MUC2 and ZO1, with enlarged pictures of the section from enclosed areas with square, in the colon of the experimental mice. Bars, 100 μm. (**d**) Data for quantitative analysis of immunohistochemistry were expressed as box-and-whisker plots with median values and 10, 25, 75, and 90 percentiles. Asterisk indicates statistically significant differences compared to AOM/DSS-treated control group; *P* < 0.05. Ctrl, control. Nel-A, nelumal A.

The relative mRNA expression levels of the genes *Cox2*, *Cyclind1*, *Inos*, *Pcna*, *Muc2*, and *Tjp1* in colonic mucosa by qRT-PCR analyses are shown in Fig. 3a,b. The expressions of *Cox2*, *Cyclind1*, *Inos*, and *Pcna* in colonic mucosa were suppressed by nelumal A administration as compared to that in the AOM/DSS group. On the other hand, the expressions of *Muc2* and *Tjp1* were upregulated by nelumal A treatment when compared to that of the AOM/DSS group. The protein expressions of MUC2 and ZO1, evaluated by immunohistochemistry, were also increased by nelumal A administration (Fig. 3c,d). These results demonstrated that treatment with nelumal A inhibited cellular proliferation and protected the intestinal barrier function in the colonic mucosa of experimental mice.

#### Effects of nelumal A on FXR signals in the ileum and FGF pathways in the liver

**Figure 4:**
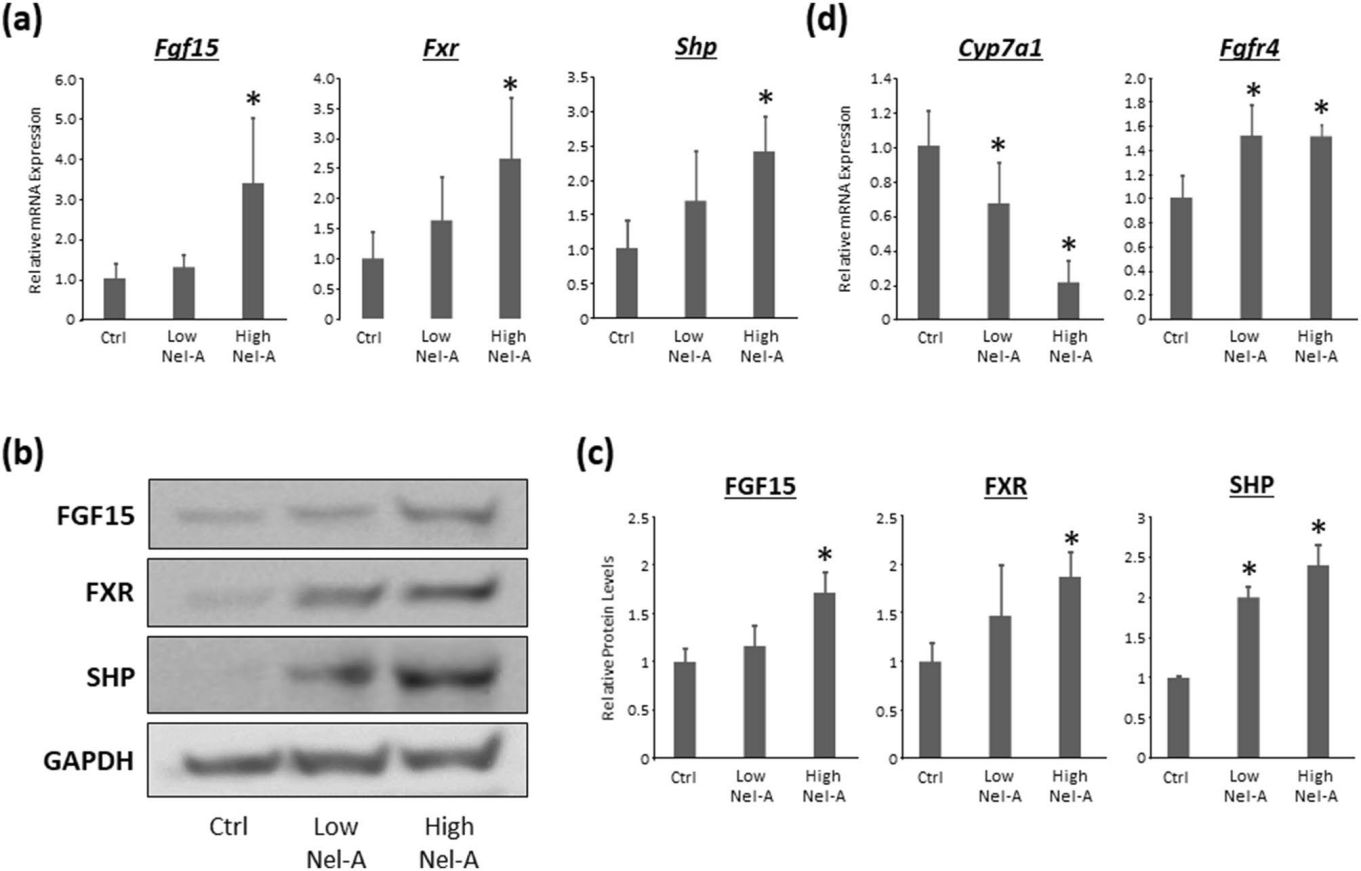
Effects of nelumal A on FXR signals in the ileum and FGF pathways in the liver in the colon of carcinogenesis model mice. (**a**) The mRNA expression levels of *Fgf15*, *Fxr*, and *Shp* in the ileum were measured by quantitative real-time reverse transcription PCR with specific primers. Parallel amplification of *18S* was used as the internal control. Statistical analyses were performed using one-way ANOVA followed by Tukey–Kramer multiple comparison test. n = 6. (**b**) Total proteins were extracted from the ileum samples of experimental mice, and protein levels of FGF15, FXR, and SHP were examined by western blot analysis using specific antibodies. GAPDH served as the loading control. (**c**) The bar graphs show the mean intensities of each protein. Statistical analyses were performed using Kruskal–Wallis test and following Steel–Dwass test. n = 4. (**d**) The mRNA expression levels of *Cyp7a1* and *Fgfr4* in the liver were measured by quantitative real-time reverse transcription as described above. Statistical analyses were performed using one-way ANOVA followed by Tukey–Kramer multiple comparison test. n = 6. Each column represents the mean ± SD. Asterisk indicates statistically significant differences compared to AOM/DSS-treated control group; *P* < 0.05. Ctrl, control. Nel-A, nelumal A.

The relative mRNA expression levels of *Fgf15*, *Fxr*, and *Shp* in the ileum were examined by qRT-PCR. Those expressions were found to be upregulated with the administration of high-dose nelumal A when compared to that in the AOM/DSS group (Fig. 4a). The protein levels of FGF15, FXR, and SHP were also investigated by western blotting and, revealing that those proteins are increased by the treatment of nelumal A (Fig. 4b,c). Next, the relative mRNA expression levels of *Cyp7a1* and *Fgfr4* in the liver were also examined by qRT-PCR. The expression of *Cyp7a1* in the liver was decreased, while the expression of *Fgfr4* increased with nelumal A treatment when compared to that in the AOM/DSS group (Fig. 4d). These results indicate that nelumal A activated FXR signaling in the intestine and inhibited de novo bile acid synthesis in the liver, which led to the attenuation of bile acid toxicity in the intestine.

## Discussions

It is well known that IBD-associated colon carcinogenesis occurs as a result of chronic inflammation. In addition, enhanced oxidative stress and increased fecal excretion of bile acids are reported to be associated with a higher risk of CRC development. As the nuclear receptor FXR regulates bile acid metabolism and may attenuate intestinal inflammation, control of this receptor signaling is expected to be a potentially effective treatment for IBD as well as a preventive approach for IBD-associated CRC. This study clearly presents the first evidence that a synthetic FXR agonist, the nelumal A, can effectively attenuate inflammation and suppress inflammation-related carcinogenesis in the colorectum of rodent models. Attenuation and suppression were thought to occur by reducing the pro-inflammatory cytokines and inhibiting oxidative stress and the subsequent cellular proliferation in the colonic mucosa. In addition, nelumal A administration decreased the proliferation and increased apoptosis in colonic tumours. Moreover, FXR signaling in the intestine was activated, and de novo bile acid synthesis in the liver was inhibited by nelumal A treatment, which might contribute to the suppression of colorectal carcinogenesis.

Chronic inflammation is a fundamental factor in the pathogenesis of cancer development in various organs including the colorectum. The present study demonstrated that nelumal A administration effectively decreased the inflammation grade in a DSS-induced colitis model. Nelumal A also prevented mucosal ulcer development and reduced the expression levels of TNF-α in the colonic mucosa in both DSS-induced colitis and AOM/DSS-induced tumourigenesis models. As TNF-α is known to be a critical tumour promoter in inflammation-related carcinogenesis^25^, the decrease in TNF-α levels by nelumal A is thought to be essential in this study. This finding is consistent with those reported in other studies in which reduction in TNF-α due to treatment with chemo-preventive agents led to the suppression of colorectal tumourigenesis^26,27^.

Enhanced oxidative stress, characterized mainly by increased reactive oxygen species, is frequently accompanied by chronic inflammation, which leads to DNA damage and consecutive carcinogenesis^28^. The effect of oxidative stress can be attenuated by local antioxidant enzymes including catalase, GPX, and SOD. In our study, mRNA expression levels of these enzymes were markedly upregulated in the colonic mucosa in the groups treated with nelumal A when compared to those in control treatment. Our results suggest that attenuation of oxidative stress might be essential for suppressing both DSS-induced colonic inflammation and AOM/DSS-induced colon carcinogenesis. Previous research has also demonstrated that decreased oxidative stress significantly suppressed colorectal tumourigenesis in rodent models^29–31^. Intestinal barrier function is also associated with inflammation and affects the intestinal condition. Disruption of the epithelial architecture causes increased mucosal permeability and detrimental intestinal conditions^32^. Administration of nelumal A markedly upregulated the expression levels of genes and proteins associated with mucosal barrier function. This alteration might protect the intestine from gut inflammation and subsequent intestinal carcinogenesis.

FXR signaling is known to regulate bile acid metabolism. It has been previously reported that secondary bile acids have harmful effects on colon mucosa through various mechanisms including inflammation, oxidative damage, and enhanced permeability. FXR activation can reduce bile acid concentrations in the intestine^13^. In this study, treatment with nelumal A upregulated the FXR-target genes *Shp* and *Fgf15* as well as proteins in the intestine. Nelumal A also upregulated *Fgfr4* and downregulated *Cyp7a1* in the liver. Our findings indicate that nelumal A served as a ligand of FXR and activated the signaling. Since CYP7A1-mediated hepatic bile acid synthesis is reduced by FXR activation, nelumal A was considered to mitigate harmful intestinal conditions such as inflammation, oxidative stress, and barrier dysfunction. The activation of FXR signaling and alteration of bile acid levels in the intestine might contribute to the suppression of intestinal inflammation and inflammation-related colon carcinogenesis.

In our study, nelumal A treatment decreased the expression levels of genes associated with cellular proliferation in the colonic mucosa, which might lead to the suppression of malignant colonic lesions. In particular, COX-2 performs fundamental roles in the growth of cancerous cells and, therefore, appears to be an essential target for CRC chemoprevention^33^. In several clinical trials, COX-2 inhibitors have been shown to possess chemo-preventive properties for CRC^34^. In addition, using murine AOM/DSS-induced tumourigenesis models, several COX-2 inhibitors were reported to inhibit colitis-related colon carcinogenesis by decreasing COX-2 expression in the colon mucosa^23,35^. The effects of nelumal A on proliferation and apoptosis in tumour tissues were also examined in this study. After nelumal A treatment, proliferation was decreased and apoptosis was increased in AOM/DSS-induced colon adenocarcinoma. The effects of nelumal A on proliferation and apoptosis might contribute to decreasing the incidence and multiplicity of adenocarcinoma by reducing the inflammation and oxidative stress in the colon mucosa as described above as well as through the direct anticancer action of the FXR agonist, which has also been previously reported^36^.

An in vivo study using nelumal A has never reported, and the present study is the first one. Since the bioavailability of nelumal A has not been investigated, the concentrations of nelumal A were determined in reference to previous reports in which auraptene, the highest dose of 500 ppm, was used as a chemopreventive agent in rodent carcinogenesis models^37,38^. Nelumal A and auraptene are considered similar agents belonging to oxyprenylated natural phenylpropanoids and functioning as FXR agonists, and the researchers have compared them regarding the activities of FXR^15^. In the report, nelumal A exhibited stronger FXR activity in comparison to auraptene. In addition, another paper indicated that nelumal A possesses more potent cytotoxicity than that of auraptene^39^. Moreover, auraptene was reported in in vivo studies to show adverse effects as antiplatelet action^40,41^. Physiological relevance to human was also assumed according to previous papers about auraptene, which is present in certain fruits, namely 0.04% in a kind of orange, 0.01–0.02% in grape fruit, and 180 μg/100 ml in grapefruit juice^42^. Due to the above considerations, the concentrations of nelumal A, similar but slightly less than that of auraptene, was determined. Regarding the stability of nelumal A, Bruyère et al.^39^ investigated in vitro and demonstrated that nelumal A was stable in physiological solution at 37℃ for 3 days, however, in vivo stability is totally unknown. In terms of cytotoxicity, a previous study demonstrated in vitro growth inhibitory activity of nelumal A with IC50 of 8–71 μM, both via cytotoxic and cytostatic effects, against various types of cancer cell lines^39^, while it appeared no cytotoxicity in the studies treating human embryonic kidney cells HEK293, human ovary granulosa-like carcinoma cells KGN, and human hepatoma cells HepG2 with 10 μM, 30 μM, and 100 μM of nelumal A, respectively^15,43^. Those findings suggest that higher levels of nelumal A may be toxic to not only cancer cells but normal cells, although this agent did not appeared to exhibit any toxicity to experimental mice in the present study. Further researches are needed to investigate the bioavailability, in vivo stability, and cytotoxicity of nelumal A.

Recent researches have indicated the relationships among gut microbiota, bile acids, and CRC development^44,45^. Primary bile acids, which are synthesized in the liver and possess important roles in cholesterol metabolism and host-microbe interaction^46^, serve as substrates for bacterial biotransformation to secondary bile acids in the large intestine. Among bile acids, secondary bile acids are considered to be mainly associated with harmful intestinal conditions, leading to colon carcinogenesis^45^. Also, it has been reported that intestinal bile acids influence the composition of gut microbiome on the contrary^47^. Namely, bile acids and gut microbiota affect each other^44^, and therefore, investigating those condition is thought essential. In the present study, however, neither the levels and components of bile acids nor the profiles of gut microbiota were investigated. In addition, other FXR agonists, such as chenodeoxycholic acid and INT-747, were not employed as controls in our study. A previous report has demonstrated that INT-747 suppresses intestinal inflammation and protect barrier function in mice colitis model^5^. Moreover, FXR-null mice was not used together in this study. Comparing with the control agents and mice might markedly help us understand the effects of nelumal A in detail. Those above, also including the bioavailability, stability, and cytotoxicity of nelumal A as described previously, are considered as limitations of the present study, therefore, further studies should be performed in order to reveal the mechanisms and potential impact of nelumal A to suppress colorectal carcinogenesis.

In conclusion, the results of the present study indicate that the synthetic FXR agonist nelumal A effectively attenuated colitis and suppressed colitis-related tumourigenesis presumably through the reduction in bile acid synthesis and decline in the inflammation and oxidative damage. These findings support the possibility that protection against gut inflammation by nelumal A may be a potentially effective and novel strategy for IBD treatment as well as IBD-related CRC chemoprevention.

## Materials and methods

### Animals and chemicals

Male A/J mice were obtained from Japan SLC (Shizuoka, Japan). All mice were housed in plastic cages under controlled conditions of humidity (50 ± 10%), light (12/12 h light/dark cycle), and temperature (23 ± 2 °C). The mice were cared for and maintained at the Gifu University Life Science Research Center (Gifu, Japan) according to the Institutional Animal Care Guidelines. The Institutional Animal Care and Use Committee of Gifu University approved all the experimental protocols and procedures used in this study (the authorization code 30-7 on 5 April 2018). AOM was obtained from Wako Pure Chemical Co. (Osaka, Japan). DSS was purchased from MP Biomedicals, LLC (Aurora, OH, USA). DSS was dissolved in distilled water at a concentration of 1.5% (w/v) to induce colitis. Dr. Francesco Epifano kindly provided the nelumal A^15^.

### Experiment 1 (DSS-induced colitis model)

A total of 25 mice were split into four experimental and one control groups and subjected to a 5-week trial. Mice in groups 1–3 received water containing DSS (1.5%) for one week. Then, they were fed the experimental diets involving 0, 100, and 400 ppm nelumal A, respectively, until the end of the study period. Mice in group 4 were fed a diet containing 400 ppm nelumal A without DSS. Mice in group 5 were not treated with DSS or nelumal A as the control. Mice in each group were euthanized and analyzed five weeks after starting the experiment. Then, weights of the body, liver, and kidneys were determined. The large intestines were flushed with physiological saline and excised. After measuring the length from the anal verge to ileocecal junction, they were cut open longitudinally along the central axis and washed with saline. The large bowels were inspected macroscopically for the existence of pathological lesions including ulcers and cuts. A histopathological examination was performed on paraffin-embedded sections from the large bowel after hematoxylin and eosin (HE) staining to determine the inflammation score in the colonic mucosa. The methods for handling the samples and scoring inflammation of the large intestine are described below. In this experiment, incidence and multiplicity of mucosal ulcer and grade of inflammation in the colon were evaluated. Also, mRNA expression levels of inflammatory cytokine *Tnfa* and anti-oxidant enzymes *Catalase* and *Gpx1* in colonic mucosa were examined.

### Experiment 2 (AOM/DSS-induced CRC model)

A total of 56 mice were divided into seven experimental and control groups. Mice in groups 1–3 were given a single intraperitoneal injection of AOM (10 mg/kg body weight). Starting one week after the AOM injection, the animals received 1.5% DSS in drinking water for seven days. Subsequently, the groups received diets containing 0, 100, or 400 ppm nelumal A for 15 weeks, respectively. Mice in group 4 were not treated with AOM or DSS and were fed a diet containing 400-ppm nelumal A. Mice in groups 5 and 6 were treated only with AOM and DSS, respectively, as described above. Mice in group 7 served as an untreated control. Animals were sacrificed at week 18 to determine the effects of nelumal A on colitis and colon tumourigenesis. The methods for handling the samples and scoring inflammation of the large intestine are described below. In the experiment 2, incidence and multiplicity of mucosal ulcer/dysplasia and adenoma/adenocarcinoma and grade of inflammation in the colon were evaluated. Cell proliferation and apoptosis in colonic adenocarcinoma were analyzed by immunohistochemistry for PCNA and cleaved caspase-3, respectively. mRNA expression levels of *Tnfa*, *Mcp1*, and *f4/80* (inflammation), *Catalase*, *Gpx1*, and *Sod1* (anti-oxidant enzymes), *Cox2*, *Cyclind1*, *Inos*, and *Pcna* (cellular proliferation), *Muc2* and *Tjp1* (barrier function) in colonic mucosa were examined. Barrier function of intestinal mucosa was also investigated by immunohistochemistry for MUC2 and ZO1. Expression levels of mRNAs associated with FXR signals (*Fgf15*, *Fxr*, and *Shp*) in the ileum and FGF pathways (*Cyp7a1* and *Fgfr4*) in the liver. Protein levels of FGF15, FXR, and SHP were also analyzed using western blotting.

### Handling and evaluating colon samples

To determine the incidence/multiplicity of the colonic lesions (mucosal ulcer and dysplasia), inflammation scores, and the mRNA expression of several markers, the colon was cut into three equal parts from the anus, and then each part was cut in half longitudinally. The half without tumours was used for mRNA expression analysis, and the remaining for pathological analysis after fixation. For pathological analysis, tissues fixed in 10% buffered formation were totally submitted as multiple transverse sections for histological processing. This averaged four pieces/tissue and 12 pieces/total colon. The colonic lesions were counted on all slides stained with hematoxylin and eosin, the sum was divided by the number of slides, and expressed as mean ± SD.

### Scoring and grading of inflammation in the large bowel

The inflammation in the large intestine was scored and graded in the HE-stained sections. Inflammation in the colon was graded and classified in accordance with the morphological criteria reported previously by Cooper et al.^48^, as follows. The grading included 0 for normal and healthy appearance, grade 1 for shortening and loss of the basal one-third of the crypts with mild mucosal inflammation, grade 2 for loss of the basal two-thirds of the crypts with moderate mucosal inflammation, grade 3 for loss of all crypts with severe mucosal and submucosal inflammation while retaining the surface epithelium, and grade 4 for the presence of mucosal ulcers with severe inflammation (infiltration of neutrophils, lymphocytes, and plasma cells) in the mucosa, submucosa, muscularis propria, and/or subserosa.

### Immunohistochemistry

Using the labeled streptavidin–biotin method (DAKO, Kyoto, Japan), immunohistochemical staining for PCNA, a marker for the G1-to-S phase, and cleaved caspase-3 was performed to examine cell proliferation and apoptosis, respectively, in the colonic tumours^26,49^. Immunohistochemical staining for MUC2 and ZO1 in the colonic mucosa was performed to evaluate mucosal barrier function. Anti-PCNA antibody (1:100, Santa Cruz Biotechnology, Dallas, TX, USA), anti-cleaved caspase-3 antibody (1:500, Ser276; Cell Signaling Technology, Danvers, MA, USA), anti-MUC2 (GeneTex, Irvine, CA, USA), and anti-ZO1 (abcam, Cambridge, UK) were used. PCNA-positive cells and cleaved caspase-3-stained cells were counted and expressed as a percentage of the total number of cells in the colonic tumours. The positive rate was determined by counting at least 200 cells in each mouse. Immunohistochemically stained areas for MUC2 and ZO1 were evaluated and quantified with NIH Image software (Bethesda, MD, USA) according to the section of tutorials and examples in the website of the software (https://imagej.nih.gov/ij/docs/examples/stained-sections/index.html). The quantitative analysis was performed with four images from each mouse in a blinded manner.

### Total RNA extraction and quantitative real time PCR

Total RNA was isolated using the RNeasy mini kit (Qiagen GmbH, Hilden, Germany) and transcribed to complementary DNA (cDNA) using the High-Capacity cDNA Reverse Transcription Kit (Applied Biosystems Japan Ltd., Tokyo, Japan). Quantitative real-time PCR analysis of individual cDNA was performed with LightCycler Nano (Roche Diagnostics, GmbH, Mannheim, Germany) and FastStart Essential DNA Green Master (Roche Diagnostics) as previously described^31^. Parallel amplification of *Gapdh* was used as an internal control. The sequences of the PCR primer pairs are shown in Supplementary Table S3.

### Protein Extraction and Western Blot Analysis

Total protein was extracted from mice ileum, and equivalent amounts of proteins (20 µg/lane) were examined by Western blot analysis^50^. Primary antibodies for FGF15 (LifeSpan BioScience, Inc., Seattle, WA, USA), FXR (abcam, Cambridge, UK), SHP (abcam), and GAPDH (Cell Signaling Technology, Danvers, MA, USA) were used for immunoblotting. The intensities of the bands were quantified with NIH Image software. GAPDH served as a loading control.

### Statistical analysis

After providing Shapiro–Wilk normality test, one-way analysis of variance (ANOVA) was used to compare the groups. If the ANOVA showed significant differences, then the Tukey–Kramer multiple comparison test was performed to compare values among the groups. Non-parametric statistical analysis was performed with Kruskal–Wallis test and following Steel–Dwass test between the groups. Fisher’s exact test was used to compare the incidence of intestinal ulcers and tumours. All data, except quantitative analysis of immunohistochemistry for MUC2 and ZO1, analyzed are presented as mean ± standard deviation (SD). All differences were considered to indicate statistical significance at a two-sided *P* value of less than 0.05.

## Supplementary Information

Supplementary Information.

## Abbreviations

ANOVA: Analysis of variance;
AOM: Azoxymetahne
CRC: Colorectal cancer
DSS: Dextran sodium sulfate
FXR: Farnesoid X receptor
Gpx: Glutathione peroxidase
HE: Hematoxylin and eosin
Mcp: Monocyte chemoattractant protein
PCNA: Proliferating cell nuclear antigen
qRT-PCR: Quantitative transcription PCR
SD: Standard deviation
TNF: Tumour necrosis factor
