# Supplementary material for: Novel FXR agonist nelumal A suppresses colitis and inflammation-related colorectal carcinogenesis
Source: Sci Rep. 2021 Jan 12;11:492. doi: 10.1038/s41598-020-79916-5 (PMC7804240; doi:10.1038/s41598-020-79916-5)

**Novel FXR agonist nelumal A suppresses colitis and inflammation-related colorectal carcinogenesis**

Tsuneyuki Miyazaki[1]†, Yohei Shirakami[1]†*, Taku Mizutani[1], Akinori Maruta[1], Takayasu Ideta[1], Masaya Kubota[1], Hiroyasu Sakai[1], Takashi Ibuka[1], Salvatore Genovese[2], Serena Fiorito[2], Vito Alessandro Taddeo[2], Francesco Epifano[2], Takuji Tanaka[3], Masahito Shimizu[1]

[1]Department of Gastroenterology, Gifu University Graduate School of Medicine, Gifu 501-1194, Japan.

[2]Department of Pharmacy, D'Annunzio University of Chieti–Pescara, 66100 Chieti Scalo, Italy.

[3]Department of Pathological Diagnosis, Gifu Municipal Hospital, Gifu 500-8513, Japan.

†These authors are equally contributed to this work.

*Correspondence to: Yohei Shirakami, Department of Gastroenterology, Gifu University Graduate School of Medicine, 1-1 Yanagido, Gifu 501-1194, Japan. E-mail: ys2443@gifu-u.ac.jp

**Supplementary Table S1.** General observation in the experiment 1.

| Group No. | Treatment | No. of mice | Body weight (g) | Relative liver weight (g/100 g body weight) | Length of the large bowel (cm) |
|---|---|---|---|---|---|
| G1-1 | DSS | 5 | 23.6 ± 2.0[a] | 5.0 ± 0.2 | 14.8 ± 0.8 |
| G1-2 | DSS/100 ppm nelumal A | 5 | 24.6 ± 1.8 | 4.8 ± 0.2 | 14.1 ± 1.3 |
| G1-3 | DSS/400 ppm nelumal A | 5 | 24.6 ± 1.5 | 5.0 ± 0.1 | 13.4 ± 1.3 |
| G1-4 | 400 ppm nelumal A | 5 | 23.7 ± 1.7 | 4.7 ± 0.2 | 13.5 ± 0.9 |
| G1-5 | No treatment | 5 | 24.1 ± 0.7 | 5.0 ± 0.3 | 14.6 ± 0.4 |

[a] Deta are given as the mean ± SD.

**Supplementary Table S2.** General observation in the experiment 2.

| Group No. | Treatment | No. of mice | Body weight (g) | Relative liver weight (g/100 g body weight) | Length of the large bowel (cm) | Inflammation grade in colon mucosa |
|---|---|---|---|---|---|---|
| G2-1 | AOM/DSS | 12 | 27.3 ± 3.1[a] | 4.8 ± 0.2 | 13.7 ± 0.9 | 3.9 ± 0.3 |
| G2-2 | AOM/DSS/100 ppm nelumal A | 12 | 27.8 ± 3.0 | 4.5 ± 0.2 | 13.0 ± 1.5 | 2.8 ± 1.9 |
| G2-3 | AOM/DSS/400 ppm nelumal A | 12 | 28.2 ± 2.8 | 4.3 ± 0.2 | 12.4 ± 1.8 | 2.6 ± 1.6[b] |
| G2-4 | 400 ppm nelumal A | 5 | 28.2 ± 1.7 | 4.3 ± 0.4 | 13.8 ± 1.6 | 0.0 ± 0.0 |
| G2-5 | AOM alone | 5 | 26.7 ± 0.7 | 4.1 ± 0.3 | 14.1 ± 1.7 | 0.0 ± 0.0 |
| G2-6 | DSS alone | 5 | 25.9 ± 1.0 | 4.4 ± 0.2 | 14.3 ± 0.5 | 0.4 ± 0.5 |
| G2-7 | No treatment | 5 | 27.1 ± 2.3 | 4.2 ± 0.3 | 15.2 ± 0.3 | 0.0 ± 0.0 |

[a] Deta are given as the mean ± SD .

[b] Significantly different from group 1 by Tukey-Kramer multiple comparison test ($P < 0.05$).

**Supplementary Table S3.** Primer sequences

| Target gene | forward | reverse |
|---|---|---|
| *Catalase* | CCTCCTCGTTCAGGATGTGGTT | CGAGGGTCACGAACTGTGTCAG |
| *Cox2* | GAAGTCTTTGGTCTGGTGCCT | GCTCCTGCTTGAGTATGTCG |
| *Cyp7a1* | AGCAACTAAACAACCTGCCAGTACTA | GTCCGGATATTCAAGGATGCA |
| *F4/80* | ACAAGACTGACAACCAGACGG | TAGCATCCAGAAGAAGCAGGCGA |
| *Fgf15* | GACTGCGAGGAGGACCAAAA | CAGCCCGTATATCTTGCCGT |
| *Fgfr4* | GCCTCCGACAAGGATTTGGCA | GAGTGCAGACACCCAGCAGGT |
| *Fxr* | CGAATCCTCCTCATGGCCTC | TCCCATGATAGGGCGGAAGA |
| *Gapdh* | GACATCAAGAAGGTGGTGAAGCAG | ATACCAGGAAATGAGCTTGACAAA |
| *Gpx1* | TTTCCCGTGCAATCAGTTC | TCGGACGTACTTGAGGGAAT |
| *Il-6* | CCGGAGAGGAGACTTCACAGAG | CTGCAAGTGCATCATCGTTGTT |
| *Inos* | CGAAACGCTTCACTTCCAA | TGAGCCTATATTGCTGTGGCT |
| *Mcp1* | TTAAAAACCTGGATCGGAACCAA | GCATTAGCTTCAGATTTACGGGT |
| *Muc2* | GGGAGGGTGGAAGTGGCATTGT | TGCTGGGGTTTTTGTGAATCTC |
| *Shp* | AGCTGGGTCCCAAGGAGTAT | CTTGAGGGTAGAGGCCATGA |
| *Sod1* | CAGGACCTCATTTTAATCCTCAC | TGCCCAGGTCTCCAACAT |
| *Tjp1* | GCACCATGCCTAAAGCTGTC | ACTCAACACACCACCATTGC |
| *TNF-α* | TGGCCCAGACCCTCACACTCAG | ACCCATCGGCTGGCACCACT |

## Figure S1

(a)

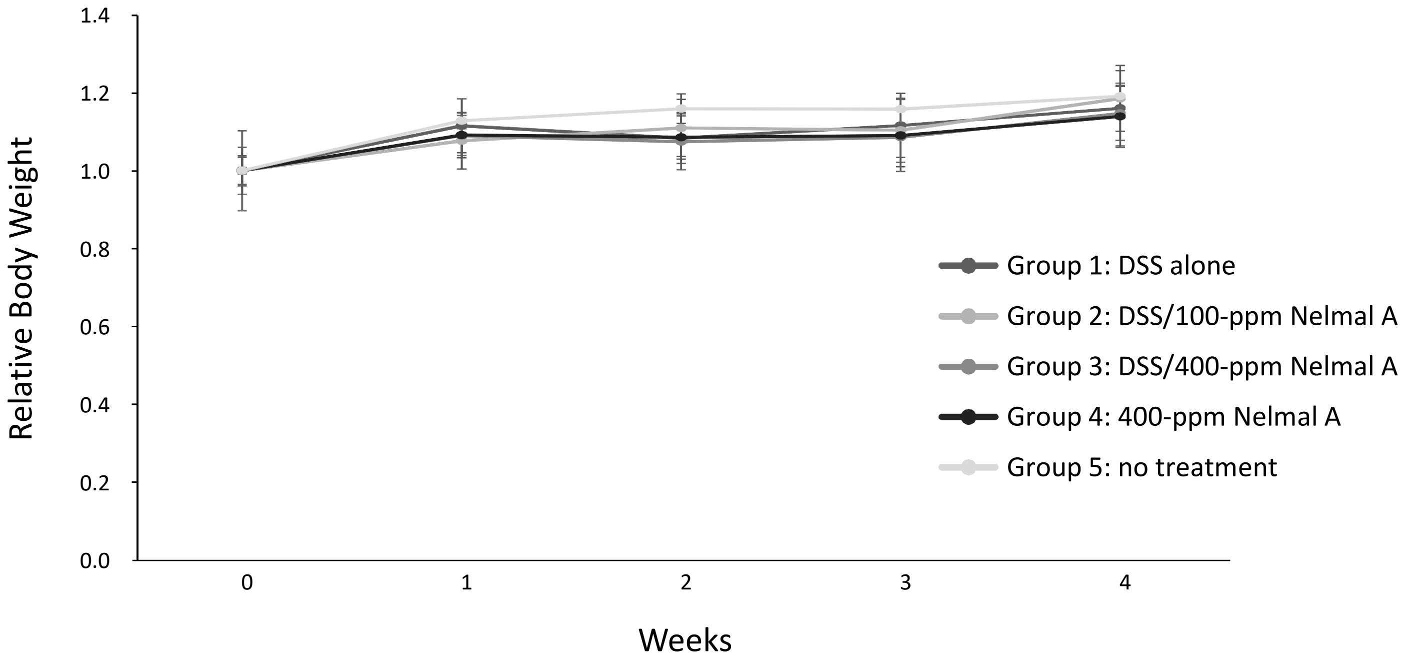

1.4
1.2
1.0
0.8
0.6
0.4
0.2
0.0
Relative Body Weight
0
1
2
3
4
Weeks
Group 1: DSS alone
Group 2: DSS/100-ppm Nelmal A
Group 3: DSS/400-ppm Nelmal A
Group 4: 400-ppm Nelmal A
Group 5: no treatment


(b)

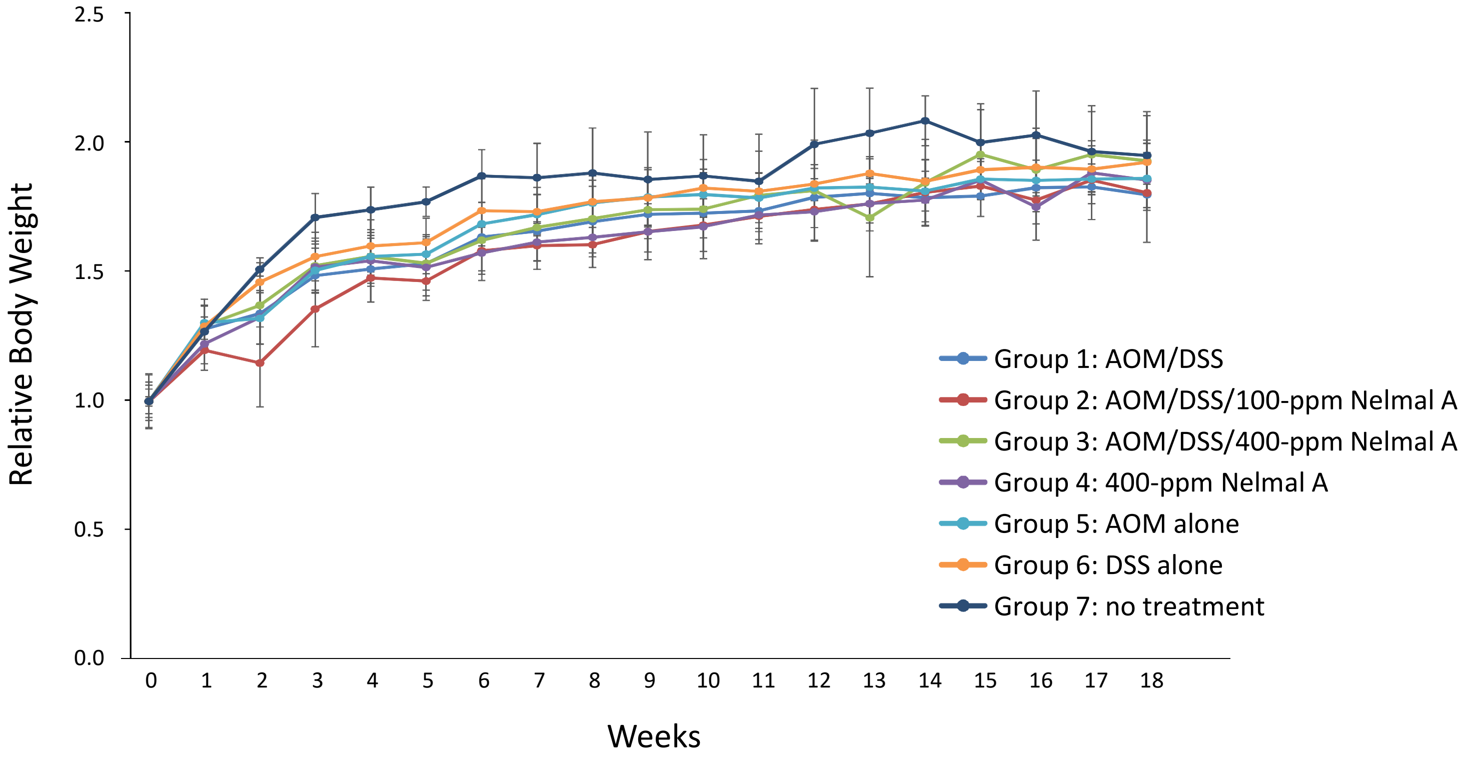

2.5
2.0
1.5
1.0
0.5
0.0
Relative Body Weight
0
1
2
3
4
5
6
7
8
9
10
11
12
13
14
15
16
17
18
Weeks
Group 1: AOM/DSS
Group 2: AOM/DSS/100-ppm Nelmal A
Group 3: AOM/DSS/400-ppm Nelmal A
Group 4: 400-ppm Nelmal A
Group 5: AOM alone
Group 6: DSS alone
Group 7: no treatment

**Figure S2**

Uncropped blots for main figure (Figure 4b)

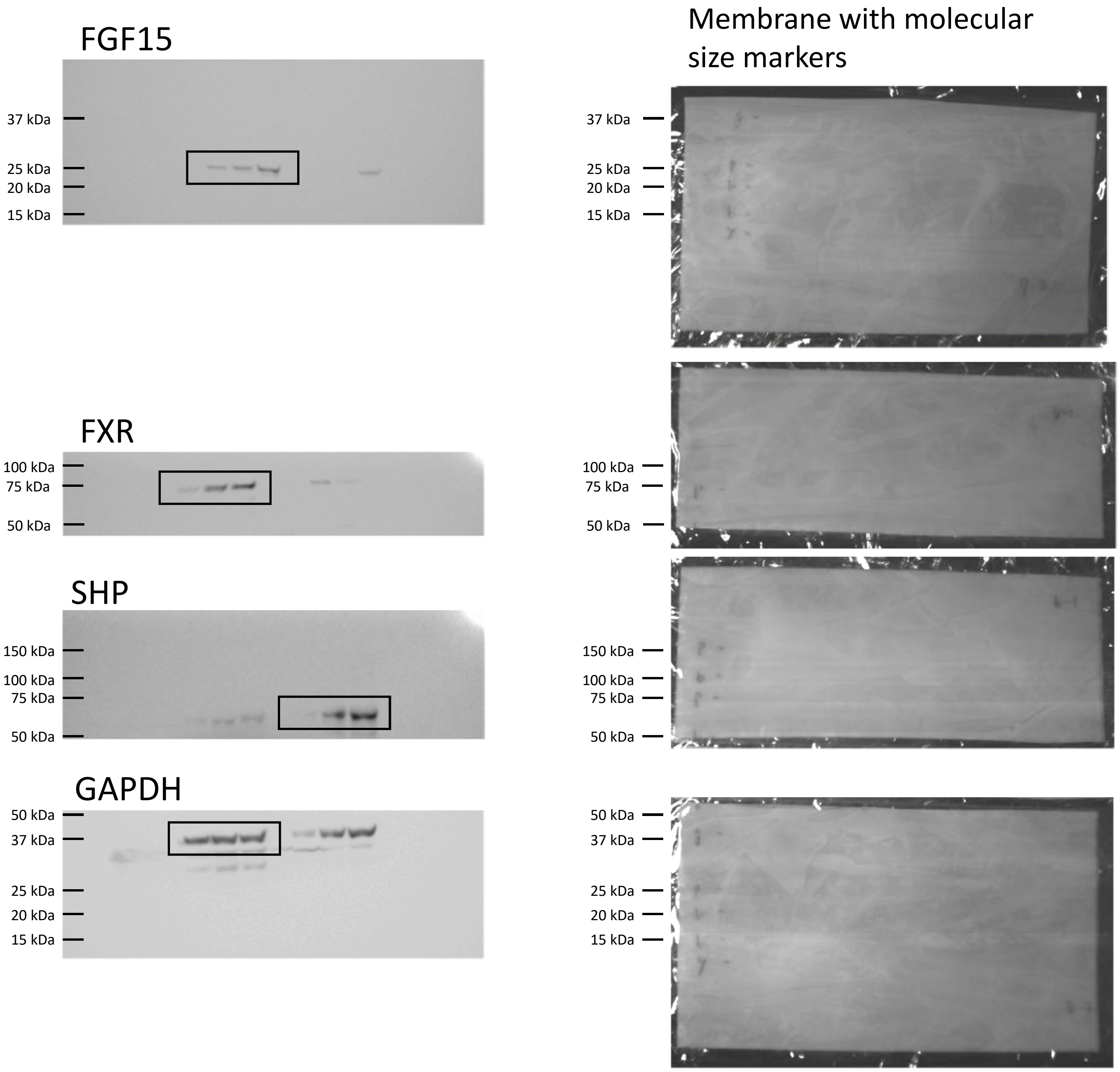

Supplement: Supplementary file 1 — Supplementary Information. [file 41598_2020_79916_MOESM1_ESM.pdf]
